# Lyophilized Emulsions in the Form of 3D Porous Matrices as a Novel Material for Topical Application

**DOI:** 10.3390/ma14040950

**Published:** 2021-02-17

**Authors:** Weronika Prus-Walendziak, Justyna Kozlowska

**Affiliations:** Faculty of Chemistry, Nicolaus Copernicus University in Torun, Gagarina 7, 87-100 Torun, Poland; weronika.pw@doktorant.umk.pl

**Keywords:** freeze-dried emulsion, porous matrices, encapsulation, microparticles, *Calendula officinalis* flower extract

## Abstract

Researchers are constantly searching for innovations that can be applied to the cosmetic industry. Production of porous materials stored in a lyophilized form and swollen directly before use may be beneficial considering their facilitated packaging, transport and storage. In this study, we propose porous materials based on sodium alginate, gelatin, glycerol and lipids (cottonseed oil and beeswax) obtained by freeze-drying and cross-linking. Material composition with the most promising properties was modified by the addition of PLA microparticles with *Calendula officinalis* flower extract. The structure and properties of obtained porous materials were analyzed. ATR-FTIR, mechanical properties, residual moisture content, porosity and density were assessed, as well as swelling properties and degradation after their cross-linking. The loading capacity and in vitro release of *Calendula officinalis* flower extract were performed for samples with incorporated PLA microparticles containing plant extract. The modification of the composition and fabrication method of materials significantly influenced their physicochemical properties. The selected plant extract was successfully incorporated into polymeric microparticles that were subsequently added into developed materials. Prepared materials may be considered during designing new cosmetic formulations.

## 1. Introduction

Freeze-drying, otherwise called lyophilization, is a dehydration method based on water removal by sublimation under reduced temperature and at low pressure [1]. This method is widely used to prepare porous three-dimensional materials because the frozen water is sublimed into the gas phase, which results in pore formation. These materials found many applications, particularly in medicine for tissue engineering due to the microenvironment they provide for the cells’ adsorption and proliferation or growth factors incorporation in order to improve regeneration of damaged tissues and organs [2,3,4]. However, the porous structure of materials is also beneficial for wound healing—the dressing may be a breathable protective barrier that maintains the optimal microenvironment for the wound healing process, as well may constitute a micro-skeleton for the migrating cells involved in granulation and epithelialization [5,6,7,8]. Various biopolymers may be employed in the preparation of porous materials, such as collagen [9,10], gelatin [11,12], chitosan [13], fibroin [14], sodium alginate [15], pullulan [16] and cellulose [17].

The addition of oils to water in the presence of an emulsifier (which lowers the surface tension between two phases) leads to the creation of an emulsion system, which consists of two immiscible phases: aqueous and oily. Due to the enhancement of the therapeutic effect on the skin, emulsions found their main application in the cosmetic industry. However, emulsions may be thermodynamically unstable systems due to the high content of water, which can also promote microbial growth. Moreover, in emulsions may occur many undesirable reactions between the ingredients of the formulation leading to a decrease in their therapeutic effect. The emulsions stability decreases with storage time, which determines the shelf life of the products and therefore limits their application in the industry. Furthermore, packaging, storage and transport of products increases the cost of produced emulsions. Freeze-drying of the emulsion may be a solution to overcome these drawbacks [18]. As a result of emulsion lyophilization, anhydrous or almost anhydrous, light and porous products are obtained. Lyophilized emulsions combine the advantages of both emulsions and freeze-dried forms. Compared to conventional emulsions, lyophilized emulsions exhibit several technological assets: increased shelf life, facilitated and lower-cost transport, as well as lack of bacteriological growth due to the very low water activity. There are few reports on the design of materials as a result of lyophilization of emulsions, mainly for medical applications as a clip application to clamping weak objects [19], for tissue engineering [20,21], but also as an absorbent of organic liquids [22].

Properties of this kind of materials may be further enhanced by the addition of the microparticles. They are spherical particles with a diameter of 1 to 1000 µm composed of active substances enclosed in the matrix composed of polymers, proteins or lipids [23]. Among many various substances that can be incorporated in microparticles, we can distinguish plant extracts [24,25]. Microparticulate system enables to isolate and protect extracts that are sensitive to temperature, pH, light or oxidation, improve their physicochemical properties, as well as control and modify their release [26]. These advantages of encapsulation results in enhanced effectiveness of plant extracts. One of the extracts showing a strong therapeutic effect on the skin is *Calendula officinalis* flower extract due to its complex composition containing polyphenols, carotenes, terpenoids, tocopherols, carbohydrates, lipids, quinines, amino acids, resins and minerals [27,28]. In our previous studies, we modified collagen-based porous matrices by adding the microspheres based on collagen and gelatin [29,30], xanthan and gellan gum [31], as well as poly(vinyl alcohol) [32]. However, freeze-dried emulsion presents a new approach in the preparation of materials indented for cosmetic chemistry.

To the best of our knowledge, there are no reports on the porous materials obtained by freeze-drying and cross-linking of emulsions intended for cosmetic purposes. Furthermore, the incorporation of microparticles into such materials has not yet been investigated.

The aim of this research was the preparation and characterization of lyophilized emulsions. Materials were based on biopolymers (sodium alginate and gelatin), plasticizer (glycerol) and lipids (cottonseed oil and beeswax). Matrices were subjected to cross-linking and re-lyophilization. We assessed their structure and properties: ATR-FTIR, mechanical, swelling, degradation, residual moisture content, porosity and density. PLA microparticles with *Calendula officinalis* flower extract were added to the material with the most promising properties in terms of cosmetic chemistry. That enabled the fabrication of porous matrices that may protect enclosed plant extract and control its release.

## 2. Materials and Methods

### 2.1. Materials

Gelatin from porcine skin (type A), poly (vinyl alcohol), Span 80 and beeswax were purchased from Sigma-Aldrich (Poznan, Poland). Polylactide was supplied from NatureWorks (Minnetonka, MN, USA). Sodium alginate was obtained from BÜCHI Labortechnik AG (Flawil, Switzerland). Glycerol was purchased from Avantor Performance Materials Poland S.A. (Gliwice, Poland). Cottonseed oil was supplied by Alston Nova, S.L. (Barcelona, Spain). The hydroglycolic *Calendula officinalis* flower extract (propylene glycol/water (80:20)) was obtained from Provital S.A. (Barcelona, Spain). All used chemicals were of analytical grade.

### 2.2. Methods

#### 2.2.1. Preparation of Polylactide Microparticles

The emulsion/solvent evaporation method was employed to obtain polylactide (PLA) microparticles with *Calendula officinalis* flower extract. In the first place, a 5% (*w*/*v*) of polylactide solution in dichloromethane (DCM) was prepared. Meantime, PVA was dissolved in 0.1% hydroglycolic *Calendula officinalis* flower extract at a concentration of 1% (*w*/*v*). Subsequently, the organic phase containing PLA was slowly added to the PVA solution over five minutes under magnetic stirring at 800 rpm. The volume ratio of PLA:PVA was 1:10. Afterward, DCM was evaporated by continuous stirring for four hours at room temperature. The *Calendula officinalis* flower extract-loaded PLA microparticles were collected by centrifugation at 10,000 rpm for 10 min. Removal of residual PVA was carried out by triple washing the microspheres with deionized water and centrifugation.

#### 2.2.2. Preparation of Freeze-Dried Emulsion Matrices

In the beginning, two mixtures were made: aqueous phase containing sodium alginate (ALG), gelatin (GEL) and different amounts of glycerol (G) and an oily phase consisting of cottonseed oil, beeswax and emulsifier Span-80 according to the proportions presented in Table 1. Both phases were heated (70–80 ℃), and subsequently mixed and homogenized (20,000 rpm, 3 min) (T25 digital ULTRA-TURRAX disperser, IKA Werke, Staufen, Germany). The solutions were cast on glass plates that were subsequently frozen (−20 ℃) and lyophilized (−55 °C, 5 Pa, 24 h) (ALPHA 1−2 LD plus lyophilizator, Martin Christ, Osterode am Harz, Germany). Afterward, the samples were cross-linked by immersion in 0.5 M CaCl_2_ for 10 min, rinsed three times with deionized water, re-frozen (−20 ℃) and re-lyophilized (−55 °C, 5 Pa, 24 h). Moreover, the control samples, which did not contain the oily phase, were also prepared.

After all, the samples were characterized; one sample with the highest potential in terms of cosmetic chemistry was selected to which PLA microparticles were added. Material containing 1% of glycerol and 3.4% of lipids was chosen due to its satisfactory visual, organoleptic and application properties. In order to obtain a microparticles-loaded emulsion matrix, the PLA microspheres containing *Calendula officinalis* flower extract were added to the solution before casting.

**Figure 1:**
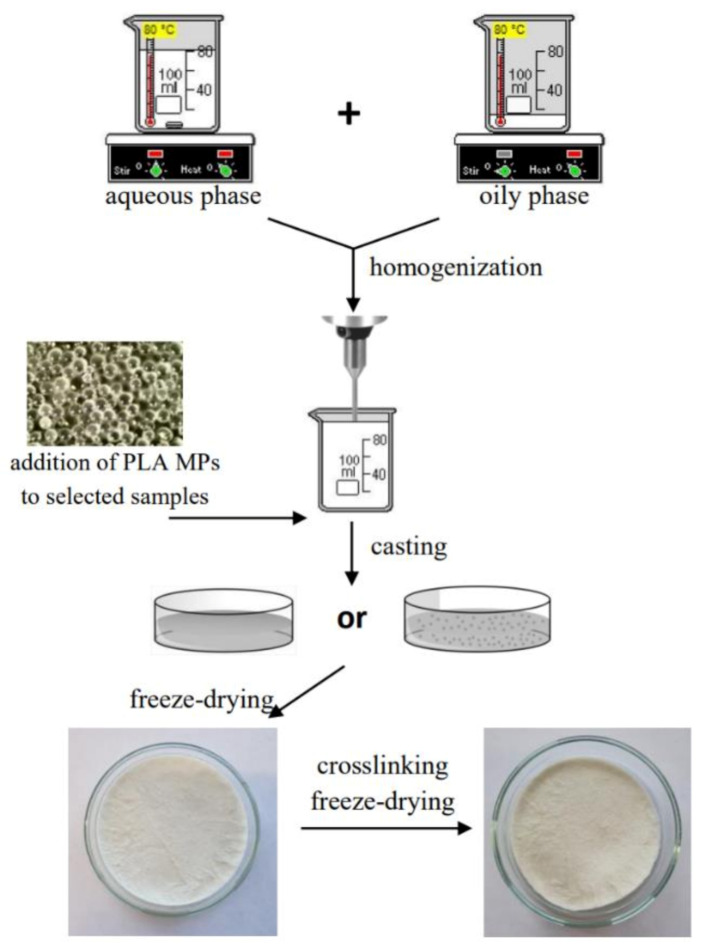
The scheme of preparation of freeze-dried emulsion matrices. Adapted from [33].

The scheme of preparation of materials is presented in Figure 1.

#### 2.2.3. Characterization of Materials

##### Scanning Electron Microscopy (SEM) Analysis

The examination of structure and cross-section of 3D materials was carried out by scanning electron microscopy (SEM) imaging (Quanta 3D FEG scanning electron microscope, Quorum Technologies, Lewes, UK). A thin layer of gold and palladium (SC7620 Mini Sputter Coater/Glow Discharge System, Quorum Technologies, Lewels, UK) was applied on the surface of the samples before the analysis. The diameters of PLA microspheres were measured by SEM after incorporation into materials.

##### Attenuated Total Reflection Infrared Spectroscopy (ATR-FTIR)

Attenuated total reflection infrared spectroscopy (ATR-FTIR) analysis was performed for the porous materials before cross-linking containing 1% of glycerin. The spectra were collected using Thermo Scientific Nicolet iS5 FTIR Spectrometer (Thermo Scientific, Waltham, MA, USA) equipped with an ATR device at room temperature at a resolution of 4 cm^−1^ and with 32 scans. Zinc selenide was used as an ATR crystal for which the incidence angle of the infrared radiation was 45°.

##### Moisture Content Measurements

The residual moisture content was determined as the weight loss of samples dried to a constant weight. Weighed material samples (2 cm × 2 cm) (W_w_) were dried at 105 °C for 24 h. Afterward, they have been weighed again (W_d_). All types of materials, before and after cross-linking, as well as loaded with microparticles, were subjected to this analysis. The measurements were carried out in triplicate. The moisture content, Equation (1) was calculated as the percentage of the water removed from the samples:

moisture content (%) = (W_w_−W_d_)/W_w_ · 100 (1)

##### Porosity and Density Measurements

The porosity (*Є*) and the density (*d*) of all types of obtained porous emulsions after lyophilization (both, before and after cross-linking, as well as with microparticles) were evaluated via liquid displacement [34]. The graduated cylinder was filled with isopropanol (V_1_) since it is a nonsolvent of the matrix-forming components. Weighed samples (W) were placed in the cylinder, and after 5 min, the liquid level (V_2_) was recorded. After carefully removing samples from the cylinder, the residual isopropanol volume (V_3_) was read. The porosity *Є,* Equation (2) and the density *d*, Equation (3) measurements of the matrices were performed in triplicate, and they were expressed as follows:

*Є* (%) = (V_1_ − V_3_)/(V_2_ − V_3_)·100 (2)

*d* = W/(V_2_ − V_3_) (3)

##### Swelling Properties

Swelling properties were determined by immersing weighed cross-linked porous samples (W_d_) in phosphate buffer saline (PBS, pH = 5.7) for 15 min, 30 min, 1 h, 2 h, 4 h, and 24 h. After each time, the samples were removed from the PBS solution, weighted (W_w_), and subsequently, a new portion of PBS was added to the samples. Cross-linked materials were subjected to the swelling measurements in triplicate. The swelling ratio of matrices, Equation (4) was defined as the percentage ratio of increased weight to the initial weight, as follows:

swelling ratio (%) = (W_w_ − W_d_)/W_d_·100 (4)

##### Degradation Measurements

Assessment of degradation of cross-linked lyophilized emulsions was performed by placing weighed (W_b_) samples in 24-well polystyrene plates and immersing them in PBS (pH = 5.7). The samples were incubated at room temperature for 1, 2, 3, 7, 14, 21 and 28 days. After each period, they were taken off the PBS buffer, rinsed with deionized water three times, frozen (−20 ℃), lyophilized (−55 °C, 5 Pa, 24 h) (ALPHA 1-2 LD plus freeze-dryer, Martin Christ, Osterode am Harz, Germany) and weighed again (W_a_). The test was conducted in triplicate for cross-linked matrices. The percentage weight loss, Equation (5) was calculated according to the following equation:

weight loss (%) = (W_b_ − W_a_)/W_b_·100 (5)

##### Mechanical Properties

A mechanical testing machine (Shimadzu EZ-Test EZ-SX, Kyoto, Japan) fitted with a 50 N load cell was used to test the mechanical properties of freeze-dried matrices in the form of cylindrical samples with known diameter. The measurements were performed at a compression speed of 2 mm/min. Both, lyophilized and cross-linked re-lyophilized materials were tested in dry conditions; however, matrices after cross-linking were additionally subjected to compression in a swollen form (after immersion in PBS buffer (pH = 5.7) for 1 min). Young’s modulus was calculated from the slope of the stress–strain curve in the linear region (strain from 0.2% to 0.5%). The results were recorded using the Trapezium X Texture program (version 1.4.5.) and average values of seven measurements for each type of material were calculated.

##### Loading Capacity of Materials with PLA Microparticles

The loading capacity was evaluated by quantifying the polyphenols with the use of the Folin–Ciocalteu test [35]. Weighed samples before and after cross-linking that contained PLA microspheres with *Calendula officinalis* flower extract (1 cm × 1 cm) were put into 2 mL of 1 M NaOH for 1 h, and afterward, they were centrifuged (10,000 rpm, 5 min). Subsequently, 20 µL of the supernatant solutions were mixed with 1.58 mL distilled water, 100 µL Folin–Ciocalteu reagent and, after 4 min, 300 µL saturated Na_2_CO_3_ solution. The prepared mixtures were incubated at 37 °C for 40 min until a characteristic blue color was obtained. The absorbance was measured at 725 nm using a UV–VIS spectrophotometer (UV-1800, Shimadzu, Kyoto, Japan). The presented results of polyphenol content in the materials with PLA microspheres were calculated based on the calibration curve for the standard solution (gallic acid) in the concentration range of 0–0.50 mg/mL (R = 0.9995). The results are the average of the measurements made for three samples of both types of material.

##### In Vitro Release of *Calendula Officinalis* Flower Extract

The samples (1 cm × 1 cm) of cross-linked lyophilized emulsion containing 1% of glycerol and 3.4% of lipids with incorporated PLA microparticles (ALG/GEL/G(1%)/L(3.4%) + MPs) were weighted (in triplicate) and placed in a 12-well polystyrene plate. Thereafter, 2 mL of acetate buffer (pH = 5.4) was added to each sample and they were incubated at 37 °C. The solution was collected after each hour until 7 h of incubation. The content of polyphenolic compounds was determined by the Folin–Ciocalteu test [35]. The procedure of preparing samples for the loading capacity described above was reused for the in vitro release study. The absorbance was measured at 725 nm using a UV–VIS spectrophotometer (UV-1800, Shimadzu, Kyoto, Japan). The release from the matrix containing extract-loaded microspheres was calculated based on the results of loading capacity.

## 3. Results and Discussion

### 3.1. Structure of Materials

Lyophilization of prepared solutions resulted in obtaining three-dimensional matrices. An evident difference between the samples with 1% and 10% addition of glycerol was observed. After the first freeze-drying process, materials with 1% addition of glycerol were soft and spongy, while matrices with 10% of glycerol were non-uniform, slightly elastic and stretchy. However, after cross-linking and the second freeze-drying process, the materials became harder and brittle. Samples containing 1% glycerol and 3.4% lipids (ALG/GEL/G(1%)/L(3.4%)) were selected for further modification–the addition of PLA microparticles with incorporated *Calendula officinalis* flower extract.

One can see that the matrices with a 1% addition of glycerol had a complex, porous structure with irregular macropores. On the contrary, materials with a 10% addition of glycerol were not porous. SEM images also revealed that PLA microparticles were distributed not only on the surface but also through the sample ALG/GEL/G(1%)/L(3.4%) + MPs.

**Figure 2:**
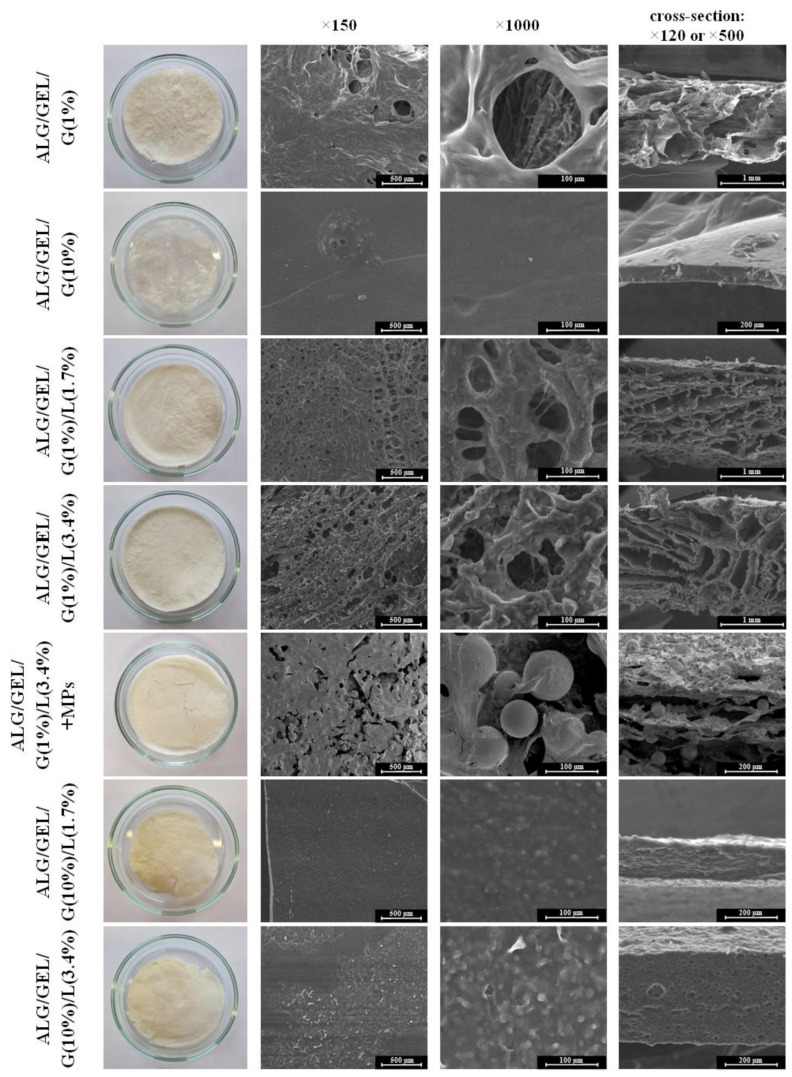
Photographs of obtained freeze-dried matrices after cross-linking and SEM images of their structure in magnification ×150 (scale bar = 500 µm), ×1000 (scale bar = 100 µm) and cross-sections in magnification ×120 (scale bar = 1 mm) or ×500 (scale bar = 200 µm).

Photographs of prepared lyophilized cross-linked materials, as well as their structure and cross-sections, are shown in Figure 2.

**Figure 3:**
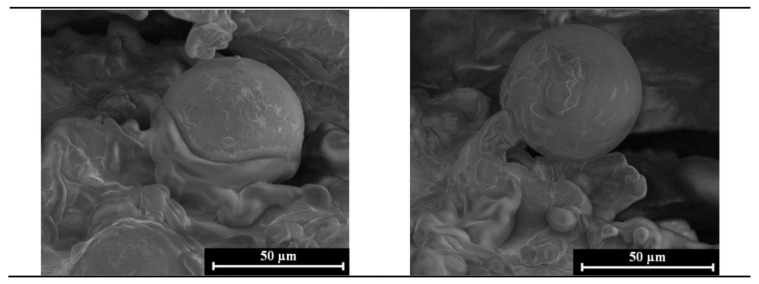
Polylactide (PLA) microparticles incorporated into porous matrix containing sodium alginate, gelatin, 1% glycerol and 3.4% lipids (ALG/GEL/G(1%)/L(3.4%) + MPs) (scale bar = 50 µm).

Figure 3 presents SEM images of polylactide (PLA) microspheres with *Calendula officinalis* flower extract incorporated into a porous matrix containing sodium alginate, gelatin, 1% glycerol and 3.4% lipids. The shape of fabricated microparticles was spherical and their surface was smooth. The diameter of the PLA microparticles was uniformly distributed (60.11 ± 10.94 µm).

### 3.2. ATR-FTIR Spectroscopy

**Figure 4:**
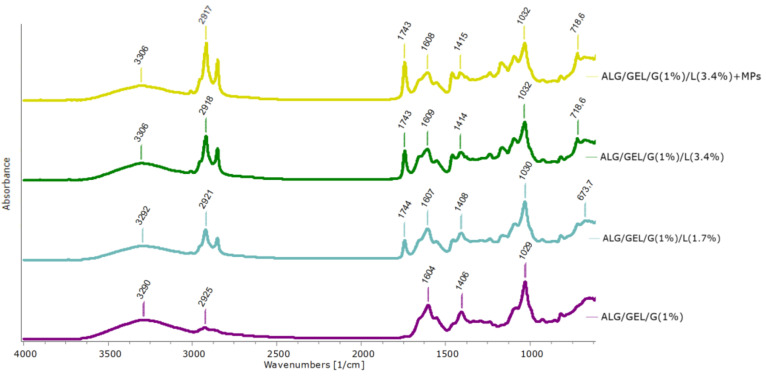
ATR-FTIR spectra of non-cross-linked materials based on sodium alginate, gelatin and 1% of glycerin, as well as with lipids (cottonseed oil and beeswax) and PLA microparticles.

ATR-FTIR spectroscopy was performed to prove the composition and functional groups of the porous materials (Figure 4).

The spectra demonstrate a broad band in the range of 3600–3100 cm^−1^ with a maximum at 3306–3290 cm^−1^. These bands were assigned to the –NH and –OH stretching vibrations (Amide A) in gelatin. Moreover, slight peaks may be seen at 3085 cm^−1^ and 2925 cm^−1^ attributed to the stretching vibrations of –NH, as well as asymmetric and symmetric –CH_2_, respectively, in the Amide B region. Other characteristic absorption bands for gelatin were noted from 1700 to 1600 cm^−1^ related to the *v*CN and *v*C = O stretching vibrations (Amide I), at 1556 cm^−1^ indicating bending *δ*NH and stretching *v*CN vibrations, as well at ~1410 cm^−1^ assigned to the symmetric *v*COO^−^(Amide II). Furthermore, a peak at 1238 cm^−1^ corresponding to the *δ*NH and *v*CN vibration of groups in Amide III was observed. The maxima corresponding to sodium alginate were noted at 1092 cm^−1^ and ~1030 cm^−1^ (attributed to the *v*C–O and *v*CO–C vibration of groups in mannuronic and guluronic units, respectively), as well as ~817 cm^−1^ assigned for *v*C–O vibration of groups in α-configuration of the guluronic units. The FTIR spectra of sodium alginate, gelatin and their mixtures are the subject of numerous studies [36,37,38], nevertheless, the materials based on these biopolymers and lipids have not been investigated. Cottonseed oil and beeswax have complex compositions that include numerous compounds. Cottonseed oil comprises mainly of unsaturated and saturated fatty acids, whereas beeswax contains higher fatty acids and long-chain alcohol esters, hydrocarbons and free acids. Therefore, the introduction of lipids into formulations leads to the signals in the region of 2960–2850 cm^−1^ belonging to the –CH_3_ and –CH_2_ of hydrocarbons and esters, as well as free fatty acids. Furthermore, a new peak emerged at ~1743 cm^−1^ corresponding to the fatty acids and long-chain alcohol esters. We also observed a small peak appearing at ~1170 cm^−1^ indicating unsaturated fatty acids. These results are in line with other studies on FTIR spectra of cottonseed oil and beeswax [39,40].

### 3.3. Moisture Content

**Figure 5:**
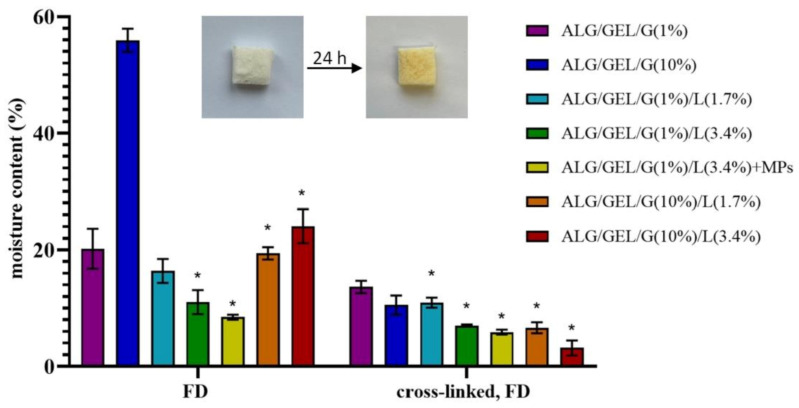
The moisture content of prepared materials: freeze-dried (FD) emulsions and cross-linked freeze-dried emulsion with different amounts of glycerol compared to the control samples (without lipids). The pictures present an exemplary sample (non-cross-linked ALG/GEL/G(1%)/L(3.4%)) before and after 24 h of drying at 105 °C. ANOVA-one way with Dunnett’s post-hoc analysis (Cl = 95%) was performed to statistically compare the results. Significant differences compared to the control for each time were marked on the graph with (*).

Figure 5 presents the results of moisture content after drying the samples, prepared by freeze-drying and cross-linking, at 105 ℃ for 24 h. The analysis demonstrated that the moisture content of samples strongly depends on the preparation method of materials. The lowest residual moisture content was observed for the samples after the cross-linking and re-lyophilization process; hence, the cross-linking of matrices led to a significant decrease in residual moisture content. The highest moisture content had samples composed of sodium alginate, gelatin and 10% glycerol obtained as a lyophilized matrix (~54%). However, the lowest residual moisture content (~3%) had cross-linked material containing 10% of glycerol and 3.4% of lipids.

The introduction of lipids into materials caused a drop in their moisture content. Another important observation is that the addition of PLA microparticles also decreased the values of sample humidity. However, the higher amount of glycerol led to a higher moisture content of samples due to the water-holding properties of glycerol.

Li et al. carried out lyophilization of bufadienolides-loaded nano-emulsion and submicro-emulsion resulting in powder [41]. They evaluated residual moisture content using thermogravimetric analysis and discovered that moisture content was approximately 4–5% which was a result of unfrozen water trapped in the sugar matrix during the sublimation drying step. They also determined that the freeze-drying conditions, such as temperature, time and vacuum, were a key factor in the amount of residual moisture content.

### 3.4. Porosity and Density Measurements

The results of porosity and density evaluated by liquid displacement of lyophilized, as well as cross-linked and re-lyophilized matrices, are shown in Table 2.

The porosity of lyophilized materials differed depending on the composition and preparation method of porous matrices. The highest porosity had a lyophilized sample comprising a lower amount of lipids and glycerol (*Є* = 80%), which indicates that the increase in the amount of lipids and glycerol contributed to the decrease in porosity. Materials containing 10% of glycerol had lower porosity than the ones with 1% glycerol. Lower porosity also characterized samples containing 3.4% of lipids in comparison to matrices with 1.7% of lipids. The effect of lipids content had a much more significant impact on the samples with 1% glycerol than 10% of glycerol. The contrary results were observed for matrices after cross-linking.

Furthermore, cross-linking and re-lyophilization led to an increase in porosity of samples containing 3.4% of lipids in their formulations and of the control sample composed of sodium alginate, gelatin and 10% glycerol. The opposite effect was noticed for samples with the addition of 1.7% of lipids and in the case of the control sample with 1% glycerol.

The incorporation of PLA microparticles into materials, regardless of their production method, increased the porosity of samples. Incorporation of microparticles into materials enhances the formation of larger pores, which is caused by their aggregation, thus reducing their ability to fill the pores [42,43]. Researches also noticed that the larger amount of particles added to the materials, the larger pores of composites [44].

Sarker et al. fabricated sodium alginate/gelatin-based lyophilized materials additionally modified by the bioactive glass for tissue engineering [45]. The porosity of sodium alginate material cross-linked by calcium chloride solution was about 70%, whereas the addition of gelatin into samples resulted in significantly lower porosity (~45%). Cuadros et al. also prepared a highly porous cross-linked alginate-gelatin matrix using the freeze-drying technique. They obtained material with a porosity of 97% and a density of 44 mg/mL [46].

Based on the presented data, it can be concluded that the composition and the fabrication method of three-dimensional matrices also affected the density of materials. The lyophilized control sample with 1% glycerol showed the lowest density (*d* = 57.8 ± 6.5 mg/mL), whereas the highest density had a lyophilized matrix with the addition of 3.4% of lipids and 10% of glycerol (*d* = 460.9 ± 2.2 mg/mL). This suggests that the increased amount of glycerol and the introduction of lipids escalated the density of materials.

The addition of PLA microparticles to lyophilized emulsion led to an increase in their density. These observations were also noticed for cross-linked and re-lyophilized materials.

Moreover, the cross-linking process increased the density of samples with a 1% addition of glycerol, whereas it decreased the density of materials containing 10% glycerol.

### 3.5. Swelling Properties

**Figure 6:**
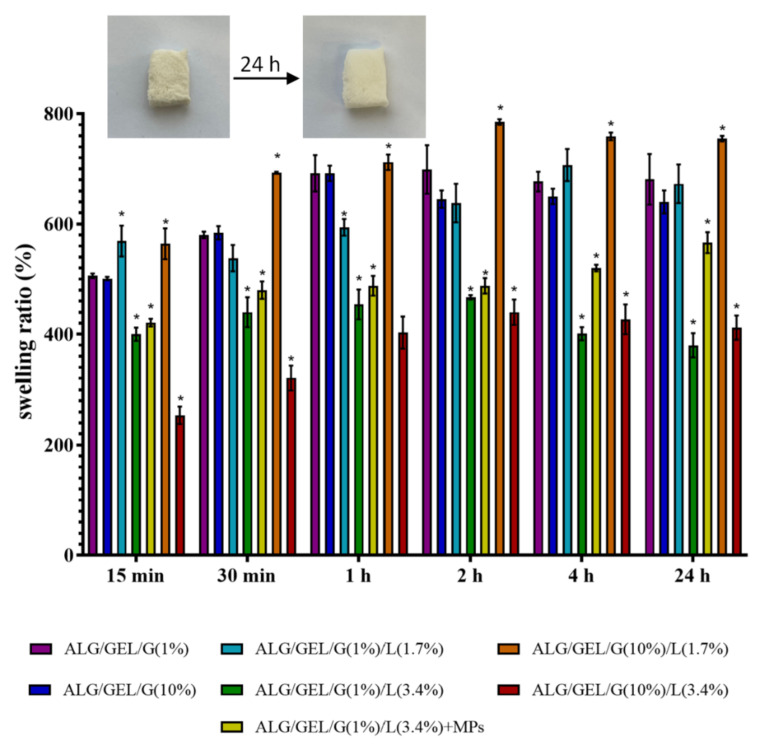
The swelling percentage of cross-linked lyophilized emulsions based on sodium alginate and gelatin with different amounts of glycerol compared to the control samples (without lipids). The pictures present an exemplary sample (cross-linked ALG/GEL/G(1%)/L(3.4%)) before and after 24 h of incubation in PBS buffer. ANOVA-one way with Dunnett’s post-hoc analysis (Cl = 95%) was performed to statistically compare the results. Significant differences compared to the control for each time were marked on the graph with (*).

The results of swelling tests of cross-linked matrices based on sodium alginate, gelatin and glycerol with the addition of lipids are shown in Figure 6. The swelling measurements were conducted after 15 min, 30 min, 1 h, 2 h, 3 h, 4 h and 24 h of incubation in PBS buffer (pH = 5.7). Freeze-dried materials (before cross-linking) dissolved rapidly after immersion in PBS buffer.

Swelling occurs when a solvent of similar polarity to porous material interacts with the surface of a material and penetrates its porous network. The swelling rate is dependent on several parameters such as porosity, drying procedure and structure of the polymer network [47].

Based on results, one can see that the control samples and the material comprising 1% of glycerol and 1.7% of lipids had similar, high swelling degrees (approximately a maximum of 700%), whereas the highest swelling degree had a sample with the addition of 10% of glycerol and 1.7% of lipids (with a maximum of 785% swelling ratio), which was also the sample with the lowest porosity. As expected, the lowest swelling properties showed samples containing 3.4% of lipids (~450%), which indicates that the higher concentration of lipids is causing lower water uptake properties.

Emulsion matrices were characterized by lower porosity and higher swelling properties. On the contrary, the control samples (without lipids) had higher porosity and thus higher swelling properties. This indicates the connection between the porosity of samples and their water uptake capacity. The research mentioned in the previous section performed by Cuadros et al. also comprised the examination of water uptake by the alginate/gelatin materials [46]. It was observed that the higher porosity of alginate/gelatin material, the higher swelling properties, which is in line with our findings. Conzatti et al. found that the swelling ability of alginate/chitosan materials is related to the hydrophilic character of alginate [48]. They also determined that the porosity is strongly related to the swelling ability of the designed materials. Especially, by introducing a macro- and microporosity, the swelling ability is strongly increased, as well as an increase in the surface area leads to higher swelling abilities of the matrices. Similarly, Pan et al. evaluated that high water uptake properties of alginate/gelatin scaffolds are probably owing to the interconnected macropores and mesopores/micropores on the surface of scaffolds [49].

Furthermore, the introduction of PLA microparticles with incorporated *Calendula officinalis* flower extract led to an increase in the swelling properties of the matrix. The maximum swelling degree for most samples (including matrices with 3.4% of lipids content, as well as the matrix containing 10% glycerol and 1.7% lipids, and the control sample with 1% glycerol) was acquired after 2 h of studies.

After 24 h of incubation in PBS buffer, the swelling ratios slightly decreased due to the degradation of the samples.

Stancu et al. developed cross-linked alginate-gelatin porous materials for bone reconstruction and evaluated their swelling properties. Their samples were divided into two groups: with the maximum swelling degree in the range of 400–550% or 650–800% depending on the alginate content. They explained it by higher hydrophilicity of the alginate, and thus, samples with a higher content of alginate exhibited superior swelling index [50].

### 3.6. Degradation Measurements

**Figure 7:**
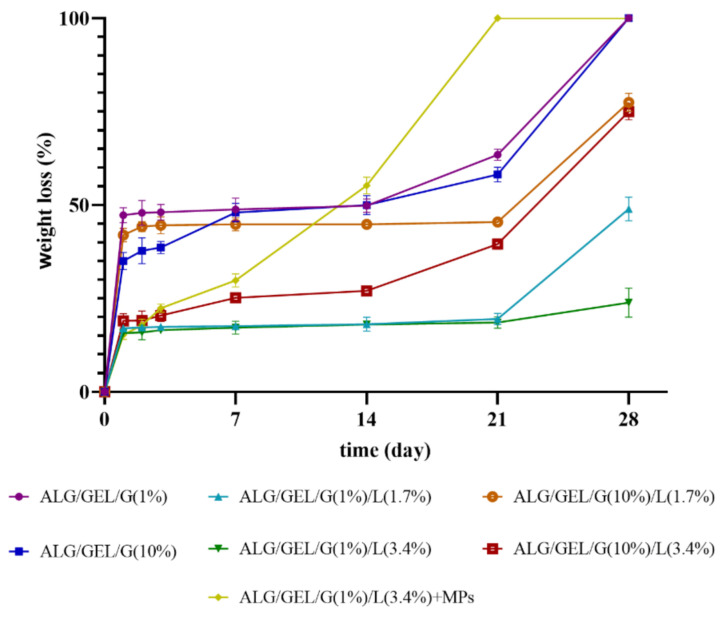
The values of weight loss during the degradation of cross-linked lyophilized emulsions based on sodium alginate and gelatin with different amounts of glycerol compared to the control samples (without lipids), as well as a sample with incorporated PLA microparticles.

The percentage weight loss of cross-linked porous matrices during their 28-days immersion in PBS buffer (pH = 5.7) is presented in Figure 7. Lyophilized materials (before cross-linking) dissolved rapidly after immersion in PBS buffer; therefore, the measurements were conducted only for materials after cross-linking.

Based on the obtained results, one can see that the weight loss of the matrix with incorporated PLA microparticles occurred the most rapidly, which suggests that the addition of microparticles significantly accelerated the degradation process of porous material. The sample with PLA microspheres fully degraded within 3 weeks.

Other samples that reached complete degradation during 4-weeks studies were the control samples (without the addition of lipids). An important observation was also that the introduction of lipids into formulations delayed their degradation. Moreover, the lyophilized emulsions containing 1% of glycerol were considerably slower to degrade than the ones with a 10% addition of glycerol. Therefore, the greatest resistance to dissolution had the sample comprising 1% glycerol and 3.4% lipids—after 28 days of incubation in PBS buffer, its weight loss was about 24%—whereas the weight loss of freeze-dried emulsions containing 10% glycerol was approximately 75%.

The weight loss of materials rocketed within the first day of immersion in medium, but after that time, it stabilized. Nevertheless, after 21-days of research, the degradation rate escalated again. Only the material containing PLA microparticles was characterized by a stable and constant weight loss after the climb during the first day of studies. It can be assumed that the rise in weight loss was related to the release of microparticles from the sample into the medium.

A similar degradation rate was observed by Wang et al. [51]. They prepared porous scaffold containing oxidized high molecular weight (HMW) and low molecular weight (LMW) alginate via freeze-drying. The degradation rate of about ~34% average weight loss by day 12 and 100% by day 39 was found as the most closely matching the natural regeneration time frame of damaged skeletal muscle.

### 3.7. Mechanical Properties

The results of Young’s modulus measurements during the compression of both types of porous matrices (after freeze-drying, as well as cross-linking and re-freeze-drying) are shown in Table 3. The cross-linked samples were measured under different conditions: dried and soaked in PBS buffer (pH = 5.7), whereas the lyophilized matrices (before cross-linking) were compressed only dried because they easily dissolved in PBS buffer.

Based on the obtained results of compression of dry lyophilized samples, we observed that a higher amount of glycerol significantly decreased the compressive modulus, and thus, these samples were more flexible. This is due to the plasticizing properties of glycerol. This effect was also noticed for cross-linked matrices.

The slighter effect on decreasing Young’s modulus (and thus stiffness of materials) had the introduction of lipids into the formulations owing to the plasticizing effect of lipids. However, an increased amount of lipids led to a slight increase in compressive modulus. The impact of the amount of lipids in the matrix compositions was much more significant in the case of cross-linked samples.

The highest Young’s modulus value was observed for the sample containing 1% glycerol and 3.4% lipids obtained by different methods: freeze-drying and cross-linking and re-freeze-drying—107.0 ± 20.9 kPa and 592.0 ± 47.8 kPa, respectively. Whereas the lowest compressive modulus exhibited samples with 10% glycerol and 1.7% lipids before cross-linking—1.8 ± 0.2 kPa, while after cross-linking—222.3 ± 32.6 kPa. Therefore, considering different preparation methods of porous matrices, the most rigid samples were ALG/GEL/G(1%)/L(3.4%), and the most flexible samples were ALG/GEL/G(10%)/L(1.7%). One can see that cross-linking of 3D matrices led to a significant increase in the stiffness of materials in dry conditions.

As expected, soaking cross-linked samples in PBS buffer caused a significant slump in compressive modulus owing to their hydration, and thus, wet samples were much less stiff. The addition and doubled amount of lipids led to a rise in Young’s modulus. In the control samples (without lipids), the higher the amount of glycerol, the lower the stiffness of the materials, whereas the opposite effect was noticed for emulsion 3D matrices, namely, the higher values of Young’s modulus were noted for the samples containing 10% of glycerol. Therefore, the highest value of compressive modulus (33.6 ± 2.7 kPa) had ALG/GEL/G(10%)/L(3.4%) sample. Glycerol and the porous structure of matrices allow the samples to quickly absorb water molecules. An observed drop in the stiffness of samples upon hydration might be caused by the weakening of hydrogen bonds within the molecular structure of the matrices.

The addition of PLA microparticles into matrices, regardless of their preparation method and the measurement conditions, caused a significant decrease in Young’s modulus. This indicates that the microparticles-loaded samples were more flexible.

The results of compressive modulus are much higher than in the previously performed studies in our team [29]. The compressive modulus of collagen/gelatin/hydroxyethyl cellulose porous matrices with collagen and gelatin microspheres was about 7 kPa in dry conditions and 0.4 kPa after immersing in PBS buffer.

### 3.8. Loading Capacity and In Vitro Release of Calendula Officinalis Flower Extract

The *Calendula officinalis* flower extract-loaded PLA microparticles were incorporated into materials based on sodium alginate and gelatin with the addition of 1% of glycerol and 3.4% of lipids. The loading capacity of these materials was examined by determining the content of polyphenolic compounds in the samples with the use of the Folin–Ciocalteu method.

We observed that the *Calendula officinalis* flower extract was successfully incorporated into freeze-dried emulsions. The preparation method of material, i.e., lyophilization and cross-linking combined with re-lyophilization, did not affect the effectiveness of the loading capacity of pot marigold flower extract. The amounts of polyphenols loaded into the freeze-dried emulsion and cross-linked freeze-dried emulsion were 81 ± 5 and 89 ± 10 mg/g, respectively.

**Figure 8:**
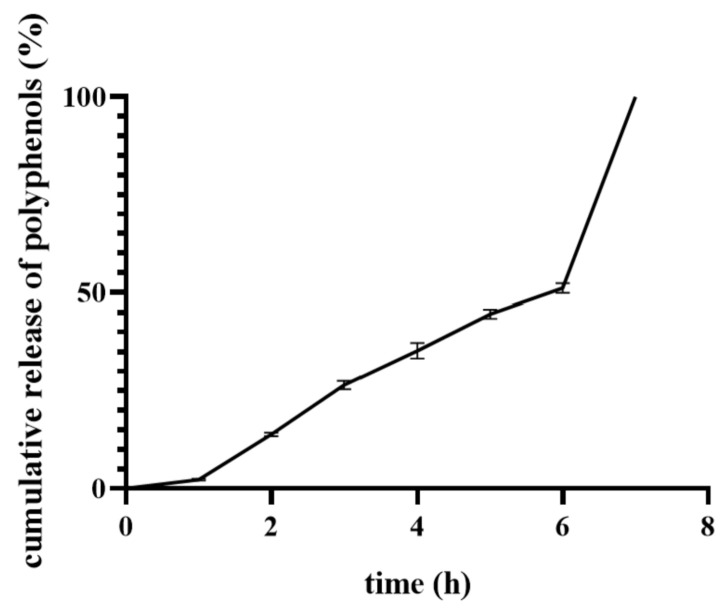
In vitro release assay of cross-linked lyophilized emulsion with incorporated PLA microspheres containing *Calendula officinalis* flower extract (ALG/GEL/G(1%)/L(3.4%) + MPs).

The *Calendula officinalis* flower extract release profile from sample based on sodium alginate and gelatin containing 1% of glycerol and 3.4% of lipids with incorporated PLA microparticles (ALG/GEL/G(1%)/L(3.4%) + MPs) in acetate buffer (pH = 5.4, 37 °C) is shown in Figure 8. Only the release from cross-linked lyophilized emulsion was tested due to the rapid solubility of lyophilized emulsion. The conditions of in vitro release assay corresponded to the skin.

One can see that the active substance enclosed in microparticles was completely released within 7 h, and it occurred in two stages. The first one lasted 6 h, and during that time, approximately 51% of loaded plant extract was released from the matrix with a relatively constant release rate. This could be associated with leaching out of microparticles from the matrix to the release medium. PLA microspheres incorporated into the material served as an extract reservoir, which prolonged the release profile of the active substance. The second stage of release was characterized by a burst release of *Calendula officinalis* flower extract. This may have been caused by the release of plant extract from microparticles by gradual diffusion of polyphenols through the polylactide matrix.

The release mechanism differs between smaller and larger microparticles. Larger microparticles mainly determine the slower release rate of the active substances due to the increase in the length of the diffusion path [52]. The fast release profile of polyphenolic compounds from smaller microparticles may be facilitated by their high surface to volume ratio [53]. Additionally, polyphenols, due to their relatively low molecular weight, can easily permeate through the polymer matrix. Therefore, their release from PLA microparticles is probably consistent with a diffusion-controlled release through the polymer matrix [54].

## 4. Conclusions

In this paper, we designed and characterized porous matrices obtained by freeze-drying and cross-linking of emulsions. Matrices based on sodium alginate and gelatin were modified by the addition of different amounts of lipids and glycerol. The introduction of lipids into material formulations led to a decrease in their moisture content, swelling ratio and degradation rate. The more glycerin, the higher the moisture content and greater flexibility but the lower the porosity as well as the stability to degradation. As a result of our research, we were able to select a sample that is most suitable for potential skin applications. The porous matrix containing 1% of glycerol and 3.4% of lipids with incorporated PLA microparticles is a very promising material for the controlled release of plant extract for dermal applications, e.g., as cosmetic masks.

## Figures and Tables

**Table 1:** The types of prepared materials composed of sodium alginate (ALG), gelatin (GEL), glycerol (G) and different amount of lipids (L): cottonseed oil, beeswax and emulsifier Span-80, as well as the sample containing polylactide microparticles (PLA MPs). Data from [33].

Sample	Composition of Materials (% *w*/*w*)						Addition (% *w*/*w*)
	Aqueous Phase			Oily Phase			PLA MPs
	ALG	GEL	G	Cottonseed Oil	Beeswax	Span-80	
ALG/GEL/G(1%)	2	1	1	–	–	–	–
ALG/GEL/G(10%)	2	1	10	–	–	–	–
ALG/GEL/G(1%)/L(1.7%)	2	1	1	1.2	0.5	0.35	–
ALG/GEL/G(1%)/L(3.4%)	2	1	1	2.4	1	0.7	–
ALG/GEL/G(1%)/L(3.4%) + MPs	2	1	1	2.4	1	0.7	6
ALG/GEL/G(10%)/L(1.7%)	2	1	10	1.2	0.5	0.35	–
ALG/GEL/G(10%)/L(3.4%)	2	1	10	2.4	1	0.7	–

**Table 2:** Porosity (*Є*) and density (*d*) of freeze-dried (FD) materials based on sodium alginate and gelatin with different amounts of glycerol and lipids, as well as samples with incorporated PLA microparticles.

Sample	Porosity (*Є*) (%)		Density (*d*) (mg/mL)	
	FD	Cross-Linked, FD	FD	Cross-Linked, FD
ALG/GEL/G(1%)	75.0 ± 0.0	66.7 ± 0.0	57.8 ± 6.5	116.3 ± 9.2
ALG/GEL/G(10%)	44.3 ± 5.2	72.2 ± 4.8	351.5 ± 1.7	151.9 ± 19.0
ALG/GEL/G(1%)/L(1.7%)	80.0 ± 0.0	41.0 ± 1.6	125.6 ± 5.0	173.6 ± 3.4
ALG/GEL/G(1%)/L(3.4%)	43.3 ± 2.9	63.3 ± 4.7	98.2 ± 6.8	137.4 ± 5.2
ALG/GEL/G(1%)/L(3.4%) + MPs	63.9 ± 2.4	64.4 ± 3.8	166.4 ± 7.9	220.0 ± 15.5
ALG/GEL/G(10%)/L(1.7%)	41.9 ± 1.6	40.0 ± 0.0	393.6 ± 11.9	318.0 ± 14.1
ALG/GEL/G(10%)/L(3.4%)	40.0 ± 0.0	59.0 ± 1.6	460.9 ± 2.2	259.4 ± 22.3

**Table 3:** The values of compressive modulus (*E_c_*) of freeze-dried (FD) and cross-linked and freeze-dried matrices based on sodium alginate and gelatin with different amounts of glycerol and lipids, as well as the sample with incorporated PLA microparticles measured under different conditions (dried and after immersion in PBS buffer).

Sample	*E_c_* (kPa)		
	FD	Cross-Linked, FD	
	Dry	Dry	Soaked in PBS
ALG/GEL/G(1%)	102.9 ± 16.4	576.3 ± 51.4	9.1 ± 0.7
ALG/GEL/G(10%)	2.0 ± 0.1	555.3 ± 27.8	7.1 ± 0.4
ALG/GEL/G(1%)/L(1.7%)	92.3 ± 12.0	317.8 ± 83.2	10.5 ± 0.9
ALG/GEL/G(1%)/L(3.4%)	107.0 ± 20.9	592.0 ± 47.8	14.3 ± 1.6
ALG/GEL/G(1%)/L(3.4%) + MPs	76.2 ± 4.7	338.0 ± 53.4	7.3 ± 0.3
ALG/GEL/G(10%)/L(1.7%)	1.8 ± 0.2	222.3 ± 32.6	14.5 ± 0.9
ALG/GEL/G(10%)/L(3.4%)	4.9 ± 0.8	465.0 ± 14.9	33.6 ± 2.7

## Data Availability

No new data were created or analyzed in this study. Data sharing is not applicable to this article.
